# Physalin F Induces Cell Apoptosis in Human Renal Carcinoma Cells by Targeting NF-kappaB and Generating Reactive Oxygen Species

**DOI:** 10.1371/journal.pone.0040727

**Published:** 2012-07-16

**Authors:** Szu-Ying Wu, Yann-Lii Leu, Ya-Ling Chang, Tian-Shung Wu, Ping-Chung Kuo, Yu-Ren Liao, Che-Ming Teng, Shiow-Lin Pan

**Affiliations:** 1 Department of Pharmacology, National Taiwan University, Taipei, Taiwan; 2 Graduate Institute of Natural Products, College of Medicine, Chang Gung University, Taoyuan, Taiwan; 3 Department of Chemistry, National Cheng Kung University, Taina, Taiwan; 4 Department of Pharmacy and Chinese Medicine Research and Development Center, China Medical University, Taichung, Taiwan; 5 Department of Pharmacy, China Medical University, Taichung, Taiwan; 6 Institute of Biotechnology and Pharmaceutical Research, National Health Research Institutes, Miaoli County, Taiwan; Wayne State University School of Medicine, United States of America

## Abstract

**Background:**

The aim of this study was to determine the molecular mechanisms of physalin F, an effective purified extract of *Physalis angulata* L. (Solanacae), in renal carcinoma A498 cells.

**Methodology/Principal Findings:**

Physalin F was observed to significantly induce cytotoxicity of three human renal carcinoma A498, ACHN, and UO-31 cells in a concentration-dependent manner; this was especially potent in A498 cells. The physalin F-induced cell apoptosis of A498 cells was characterized by MTT assay, nuclear DNA fragmentation and chromatin condensation. Using flow cytometry analysis, physalin F induced A498 cell apoptosis as demonstrated by the accumulation of the sub-G1 phase in a concentration- and time-dependent manner. Moreover, physalin F-mediated accumulation of reactive oxygen species (ROS) caused Bcl-2 family proteins, Bcl-2, and Bcl-xL degradation, which led to disruption of mitochondrial membrane potential and release of cytochrome *c* from the mitochondria into the cytosol. These effects were associated with induction of caspase-3 and caspase-9 activity, which led to poly(ADP-ribose) polymerase cleavage. However, the antioxidant *N*-acetyl-_L_-cysteine (NAC) and glutathione (GSH) resulted in the inhibition of these events and reversed physalin F-induced cell apoptosis. In addition, physalin F suppressed NF-κB activity and nuclear translocation of p65 and p50, which was reversed by NAC and GSH.

**Conclusion:**

Physalin F induced cell apoptosis through the ROS-mediated mitochondrial pathway and suppressed NF-κB activation in human renal cancer A498 cells. Thus, physalin F appears to be a promising anti-cancer agent worthy of further clinical development.

## Introduction

Among urological cancers, the incidence of renal cell carcinoma (RCC) is third in frequency after prostate and bladder cancers. In contrast to many other malignancies, RCC is generally resistant to chemotherapy, radiotherapy and hormone therapy. In addition, up to half of RCC patients will eventually develop metastatic RCC (mRCC), with a 5-year survival rate of only 9%. More recent attempts to treat this disease have used growth inhibitory and other immunotherapeutic methods such as cytokine-based regimens; however, they have had only of marginal benefit [1]. Therefore, novel approaches to the treatment of RCC are needed.

Reactive oxygen species (ROS) mediate many biological and pathological processes that are very important in normal and cancer cells. ROS include: superoxide anions (O_2_ ˙^-^), hydroxyl radicals ( ˙OH), hydrogen peroxide (H_2_O_2_), singlet oxygen (^−^O_2_), and highly reactive hydroxyl radicals. Mitochondria serve as electron transport chains that generate adenosine triphosphate (ATP) to supply energy to cells; as a result of this process, reactive oxygen species are also produced. Many prior studies have reported on natural compounds that induce the generation of ROS, directly in tumor cells and then go on to cause cell apoptosis. These natural compounds that induce the generation of ROS, could be combined with other anti-cancer drugs to potentiate their apoptotic effects. [2].

Natural products are a source of compounds that have important pharmacological activity in humans; scientists work to isolate the active ingredients from herbal medicines that may be developed into effective drugs for the treatment of human disease. Physalin F, the focus of this study, is an ethanolic extract from the plant *Physalis angulata* L. (Solanacae). *Physalis angulata* L. present in the tropics as well as sub-tropical areas, including Taiwan. *Physalis angulata* L. has been widely used to treat malaria, asthma, hepatitis, dermatitis, and for rheumatism [3]–[5]. It was already found that extracts from *Physalis angulata* L. had anticancer activity [6]–[9], but no further mechanisms of apoptosis pathway have been studied in these ten years. The active components isolated from *Physalis angulata* L. could be divided by structure as physanolide [10], withangulatin [11], physangulin [12], physalin [7], [13], and flavonol glycoside [8]. They were found to show significant biological activity. Prior reports have shown that the whole plant and organic extracts from *Physalis angulata* L. have cytotoxicity against many cancer cell lines. Hsieh *et al*., showed that extracts from *Physalis angulata* L. induced G2/M phase arrest in human breast cancer cells [14]. Wu *et al*., reported that the extracts from *Physalis angulata* L. and *Physalis peruviana* induced apoptosis in hepatoma Hep G2 cells [9]. Hsu *et al* [13] found that physalin B could induce apoptosis of melanoma cancer cells via the NOXA, caspase-3, and mitochondria-mediated pathways. Chiang *et al.* [7] found that physalin B and F, both isolated from *Physalis angulata* L., inhibited leukemic cell growth in several cell lines (K562, APM1840, HL-60, KG-1 and CTV-1), and that physalin F was found to be more potent. Fang *et al* [15] showed that the cytotoxicity of physalin B, H, and F against human and murine cancer cell lines. In addition, physalin B and F have been shown to have *in vivo* cytotoxic activity in mice inoculated with leukemic cells [6], [16] and sarcoma 180 tumours [6], [9], [11], [14], [17]. Furthermore, physalin F has been shown to have anti-inflammatory [6], [18], and immunosuppressive activity [19].

The goal of this study, was to investigate the mechanisms of physalin F associated with cell apoptosis in the renal carcinoma cells, A498. The results of this study showed that physalin F had cytotoxicity against human renal cancer cells, and this was caused by apoptosis via ROS- and NF-κB-dependent pathways.

## Materials and Methods

### Drugs and Chemicals

The plants of *Physalis angulata* L. was obtained from Tainan District Agriculture Improvement Station, Taiwan. Physalin F was extracted and purified in Professor T.S. Wu’s Lab. (Department of Chemistry, National Cheng Kung University, Taiwan). Minimum Essential Medium (MEM), RPMI 1640 medium, fetal bovine serum (FBS), penicillin, and streptomycin were obtained from Gibco BRL Life Technologies (Grand Island, NY). EGTA, EDTA, leupeptin, dithiothreitol, phenylmethylsulfonyl fluoride (PMSF), L-glutathione (GSH), *N*-acetyl-_L_-cysteine (NAC), dimethyl sulfoxide (DMSO), (±)-6-Hydroxy-2,5,7,8-tetramethylchromane-2-carboxylic acid (Trolox), (+)-sodium L-ascorbate (Vitamin C), MTT (3-[4], [5]-2,5-diphenyltetrazolium bromide), 4’-6-diamidino-2-phenylindole (DAPI), sulforhodamine B (SRB), propidium iodide (PI), and rhodamine 123 were obtained from Sigma (St Louis, MO). Antibodies to various proteins were obtained from the following sources: anti-mouse and anti-rabbit IgGs, Bcl-2, Bcl-xL, Bax, TRAIL, DR4, DR5, PPAR, NF-κB p65 antibodies were purchased from Santa Cruz Biotechnology (Santa Cruz, CA); p21, phospho-IκB-α (Ser32), phosphor-NF-κB p65 (Ser536), cleaved caspase-3, caspase-9 and 8 were purchased from Cell Signaling Technology® (Boston, MA); caspase-3 was purchased from Imgenex (San Diego, CA); cytochrome *c*, Fas, p53 and FasL were purchased from BD Biosciences (San Diego, CA); actin was purchased from CHEMICON (Temecula, CA). 2′, 7′-dichlorofluorescin diacetate (DCF-DA) was purchased from Molecular Probes (Eugene, Oregon). Nuclear Extraction kit (AY2002), electrophoretic-mobility shift assay (EMSA) kit (AY1000), and NF-κB p65 probe (5′- CATCGGAAATTTCCGGAAATTTCCGGAAATTTCCGGC-3′) were purchased from Panomics (Fremont, Calif).

### Cell Culture

The cancer cell lines A498, ACHN, and UO-31 were purchased from the American Type Culture Collection (Manassas, VA). ACHN and UO-31 were cultured in RPMI 1640 medium and A498 cells were cultured in Minimum Essential Medium. Both media were supplemented with 10% FBS (v/v) and penicillin (100 U/mL)/streptomycin (100 µg/mL). Cultures were maintained in a humidified incubator at 37°C in 5% CO_2_/95% air.

### Cytotoxicity Assay

Cells were incubated in 96-well plates (5,000 cells per well) in complete media with presence or absence of physalin F. The assay was terminated and cell survival was measured by MTT assay described in the previous study [20]. In brief, 100 µl MTT solution (0.5 mg/mL in phosphate-buffered saline; PBS) was added to each well. After 1 hr incubation at 37°C, MTT solution was removed and DMSO was added to dissolve dye. Absorbance at 550 nm was measured using a microplate reader, using MEN medium as a blank.

### Sulforhodamine B Assay

Cells were seeded into 96-well plates in medium with 5% FBS. After 24 hr, cells were fixed with 10% trichloroacetic acid (TCA) to represent cell population at the time of physalin F addition. After additional incubation of vehicle or physalin F for 48 hr, this assay was terminated and cell growth was measured by microplate reader at a wavelength of 515 nm.

### *In situ* Labeling of Apoptotic Cells

In situ detection of apoptotic cells was carried out using TUNEL apoptosis detective methods. Cells were cultured in chamber slides for 24 hr and then treated with physalin F (1, 3 and 10 µg/mL). After 24 hr treatment, cells were washed twice with PBS and fixed for 10 min with an ice-cold 1% paraformaldehyde solution. Staining was carried out according to the protocol provided by Promega (Madison, WI). Apoptosis was also assessed by nuclear morphology after staining with 4’-6-diamidino-2-phenylindole (DAPI), a DNA-binding fluorescent dye. After being fixed and permeabilized, the cells were stained with DAPI (1 µg/mL) for 15 min at room temperature. Finally, photomicrographs of TUNEL and DAPI staining were visualized and captured at ×400 magnifications with a fluorescence microscope (Nikon).

### Cell Death Detection Assay

The Roche Cell Death Detection ELISA^PLUS^ kit (Roche Diagnostics) is designed for quantitative detection of mono- and oligonucleosomal DNA fragmentation when cells are undergo apoptotic death *in vitro*. Protocol was applied from Roche and data was calculated and compared with control group.

### FACScan Flow Cytometric Analysis

After the treatment of cells with vehicle (0.1% DMSO) or compound for the indicated time courses, the cells were harvested by trypsinization, fixed with 70% (v/v) alcohol at 4C for 60 min and washed with PBS. Then, the cells were centrifuged and resuspended with 0.5 mL propidium iodide solution containing Triton X-100 (0.1%, v/v), RNase (100 µg/mL) and propidium iodide (80 µg/mL). DNA content was analyzed with the FACScan and CellQuest software (Becton Dickinson, Mountain View, CA).

### Western Blot Analysis

Total cell pellet was lysed with lysis buffer as previously described [21]. Cell homogenates were diluted with loading buffer and boiled for 10 min for detecting phosphorylation, expression, and cleavage of proteins. For Western blot analysis, proteins (30–60 µg) were separated by electrophoresis in a 10% polyacrylamide gel and transferred to a polyvinylidene fluoride membrane. After incubation at room temperature in PBS/5% nonfat milk for 1 hr, the membrane was washed three times with PBS/1% Tween 20. Then the membrane was immunoreacted with primary antibodies for overnight at 4°C. After washing with PBS/1% Tween 20, horseradish peroxidase (HRP)–conjugated goat anti-mouse or anti-rabbit IgGs were applied to the membranes for 1 hr at room temperature. Finally, the membranes were visualized with an enhanced chemiluminescence kit (Amersham, Buckinghamshire).

### Preparation of Cytosolic and Mitochondrial Fractions

After the treatment of cells with vehicle (0.1% DMSO) or compound for the indicated time courses, proteins of the cytosolic fractions were isolated using the Cytochrome *c* Releasing Apoptosis Assay Kit (Catalog #K257-100, BioVision, Inc.). The levels of cytochrome *c* in the cytosolic fractions were detected by Western blot analysis.

### Preparation of Nuclear Fractions

After cells were treated with DMSO or compound for the indicated time coursers, proteins of the nuclear fractions were isolated as previously described [22]. Briefly, after removing medium, cells were washed by PBS and resuspended in buffer A (10 mM Hepes [pH 7.9], 10 mM KCl, 1.5 mM MgCl_2_, 0.2 mM PMSF, and 0.5 mM DTT). After incubation on ice for 15 min, cells were centrifuged at 3,000 rpm for 5 min, and the supernatants were the cytosolic fraction. Then the pellets were resuspended in buffer C (20 mM Hepes, 420 mM NaCl, 25% glycerol, 1.5 mM MgCl_2_, 0.2 mM EDTA, 0.5 mM DTT, and 0.2 mM PMSF). After incubation on ice for 20 min, cells were centrifuged at 13,000 rpm for 10 min. The supernatants were nuclear extract and the level of p65/p50 were detected by Western blot analysis.

### Measurement of the Change of Mitochondrial Membrane Potential (Δψ_m_)

Cells were treated with or without the indicated agent. Thirty minutes before the termination of incubation, the rhodamine 123 solution (final concentration of 5 µM) was added to the cells and incubated for the last 30 min at 37°C. The cells were finally harvested and the mean fluorescence was determined using FACScan flow cytometric analysis.

### Measurement of reactive oxygen species (ROS)

Cells were incubated in the absence or presence of the indicated agents for 0.5, 1 or 3 hr. Thirty minutes before the termination of incubation period, DCF-DA (final concentration of 10 µM) was added to the cells and incubated for the 30 min at 37°C. Then, the cells were harvested for the detection of ROS accumulation using FACScan flow cytometric analysis.

### Electrophoretic Mobility Shift Assay (EMSA)

Cells were incubated in the absence or presence of the indicated agents for 6 hr, and nuclear extracts were prepared by Nuclear Extraction Kit (AY2002, Panomics). An EMSA kit from Panomics was utilized to determine the binding activities of NF-κB p65. Treated or untreated nuclear extracts (4 µg) were incubated with biotin-labeled p65 probe, and protein-DNA complexes were separated on polyacrylamide gel. The gel was transferred to nylon membrane and detected by using horseradish peroxidase (HRP)-based chemiluminescence. The bands were visualized after exposure to film.

### Statistical Analysis

All experiments were performed at least three times. Data were presented as the mean ± SE for the indicated number of separate experiments. Statistical analysis of data was done with Student’s *t* test. *P* values <0.05 were considered significant.

## Results

### Physalin F Inhibited Cell Viability in Human Renal Cancer Cells

**Figure 1:**
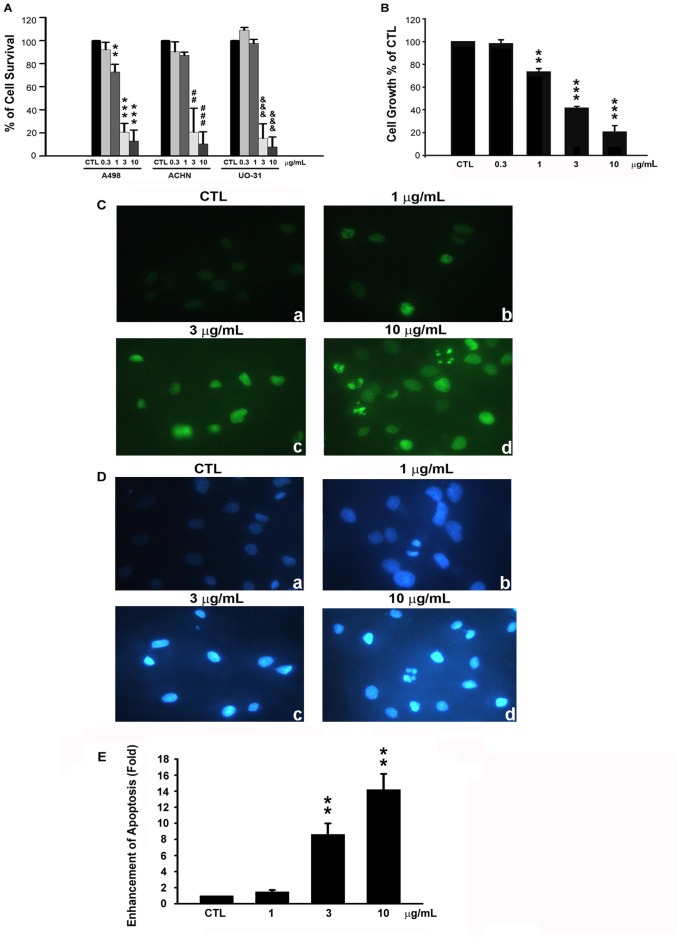
Effect of physalin F on viability in human renal cancer cells. Renal cancer cell lines (A498, ACHN, and UO-31) were treated with various concentrations (0.3, 1, 3, and 10 µg/mL) for 24 hr for MTT assay (A) and for 48 hr for SRB assay (B) in A498 cells. A498 cells were treated with vehicle or physalin F at the indicated concentration (1, 3, and 10 µg/mL) for 24 hr and viewed by fluorescence microscope followed by TUNEL (C) and DAPI (D) staining (magnification × 400). (E) To quantitatively evaluate cell death after treatment of physalin F for 24 hr, we used cell death ELISA^PLUS^ kit to detect. Data are expressed as the mean percentage of control ± S.D. of three independent experiments. **^, ##, &&^ *P*<0.01 and ***^,###, &&&^ *P*<0.001 compared with the control group.

To investigate the anti-cancer effects of physalin F, three renal carcinoma cell lines (A498, ACHN, and UO-31) were treated with various concentrations of physalin F (0, 0.3, 1, 3, and 10 µg/mL) for 24 hr and the cell viability was determined by MTT assay (Fig. 1A). Physalin F inhibited cell viability in human renal cancer cell lines A498, ACHN, and UO-31 in a concentration-dependent manner with an IC_50_ of 1.40 µg/mL, 2.18 µg/mL, and 2.81 µg/mL, respectively. However, the most potent cytotoxic effect was in A498 cells after physalin F treatment. In addition, physalin F inhibited cell growth (GI_50_ = 2.48 µg/mL) (Fig. 1B) in a concentration-dependent manner in A498 cells based on the results of the SRB assay. Also, physalin F had influences on inducing nuclear DNA fragmentation and chromatin condensation in A498 cells, according to the TUNEL assay and DAPI staining, in a concentration-dependent fashion (Fig. 1C and 1D). Finally, the results of the cell death Detection ELISA kit showed that physalin F significantly triggered A498 cell apoptosis in a concentration-dependent manner (Fig. 1E).

### Effects of Physalin F on the Cell Cycle Progression

**Figure 2:**
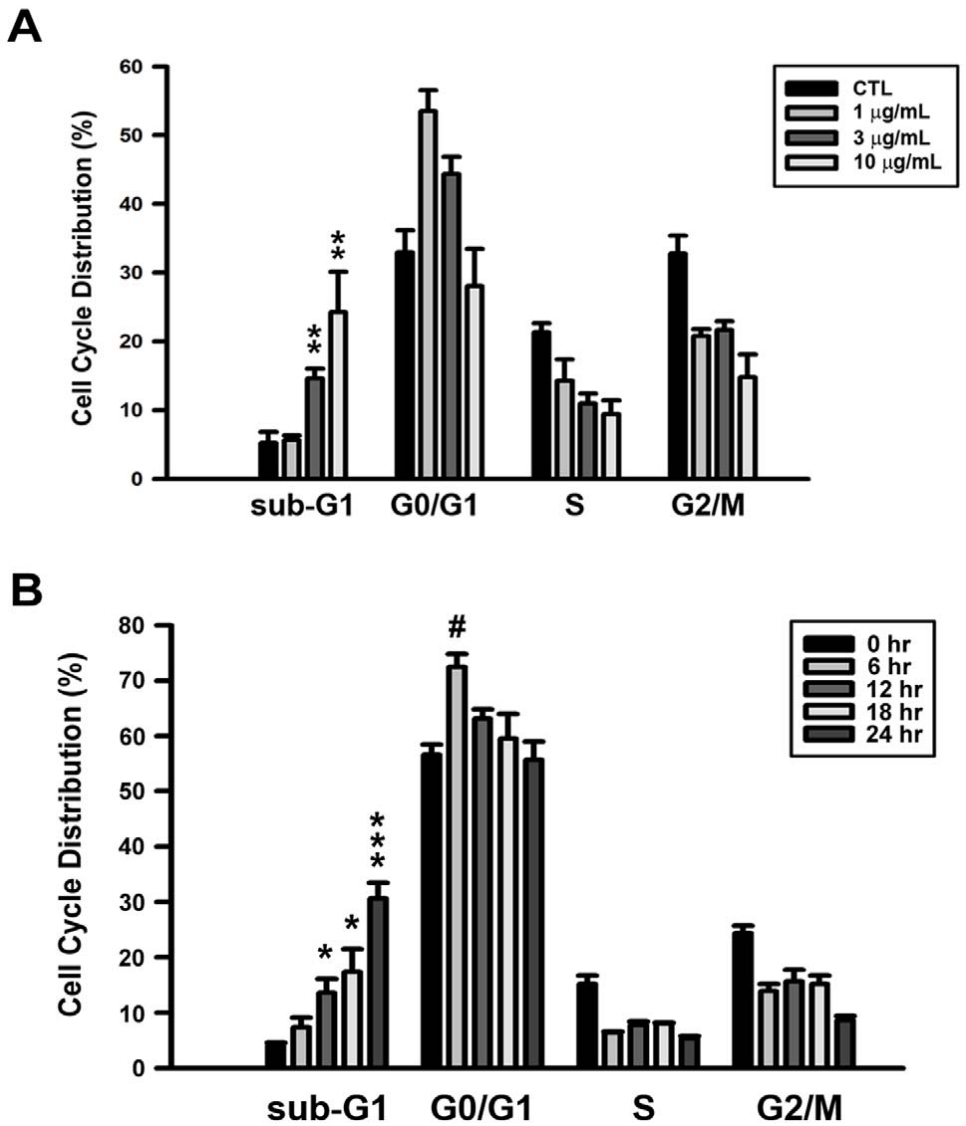
Effect of physalin F on cell cycle distribution in A498 cells. (A) Cells were incubated with vehicle or various concentrations (1, 3, and 10 µg/mL) of physalin F for 24 hr. (B) Cells were incubated with vehicle or 10 µg/mL of physalin F for indicated time period. The cell cycle analysis and cell apoptosis were determined using FACS analysis as described in Methods and Materials. * *P*<0.05, ** *P*<0.01, and *** *P*<0.001 compared with the 0-time point group in sub-G1 phase. ^#^ *P*<0.05 compared with the 0-time point group in G0/G1 phase.

The DNA cell content was measured by flow cytometry to investigate the proportion of cells in different phases of the cell cycle, after treatment with physalin F. After treatment with physalin F, the population of sub-G1 cells was increased in a concentration- and time-dependent manner (Fig. 2A and 2B). These data suggested that physalin F inhibited A498 cell viability through the apoptosis pathway.

### Physalin F Induced Cell Apoptosis via a Mitochondria-mediated Pathway

**Figure 3:**
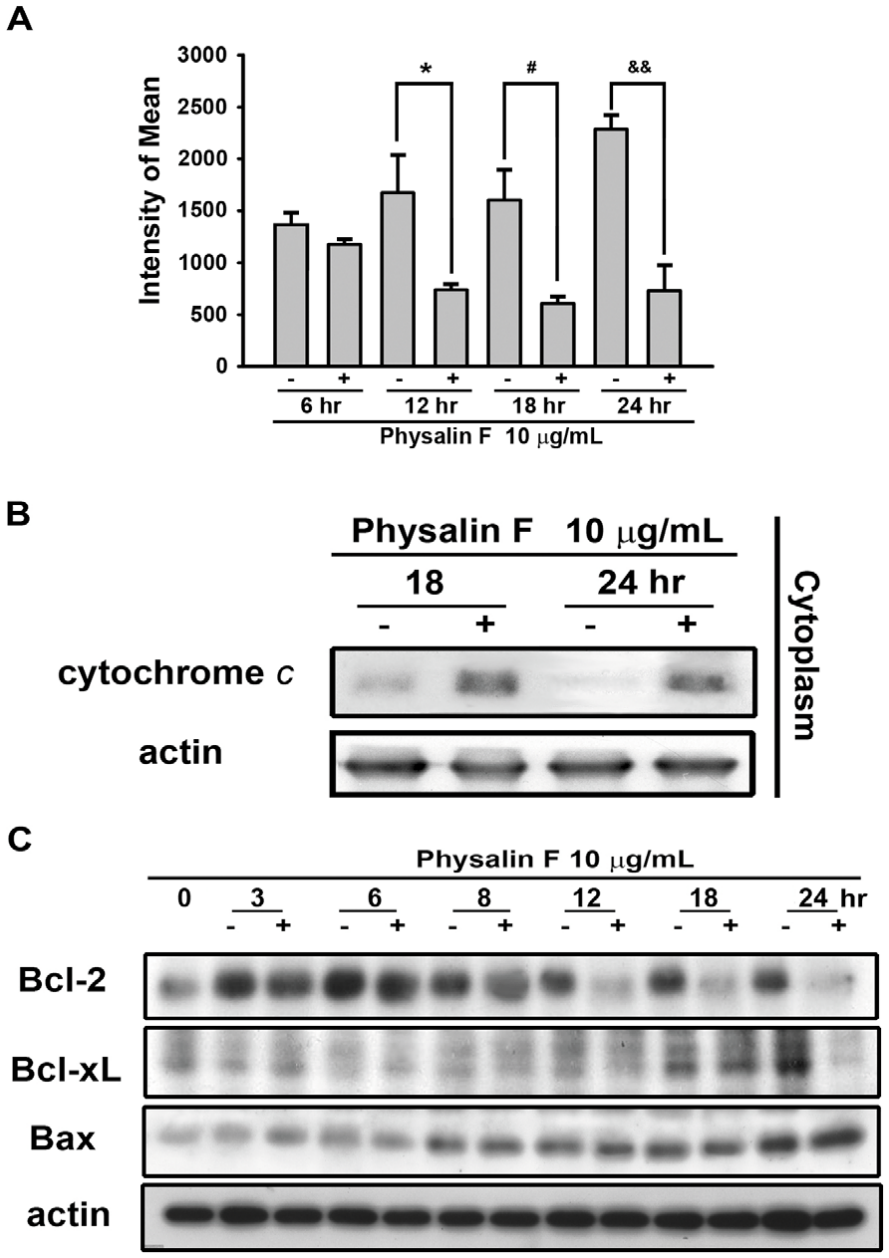
Effect of physalin F on reduction of Δψ_m_ and release of cytochrome *c*. A498 cells were incubated in the absence or presence of physalin F (10 µg/mL) for indicated time, and cells were harvested and prepared for detection (A) mitochondria membrane potential by using FACScan analysis, (B) release of cytochrome *c* in cytosol (The fractions were collected by using the protocol of preparation of cytosolic and mitochondrial fractions as mentioned in Materials and Methods.), and (C) Bcl-2, Bcl-xL and Bax expression by using Western blotting analysis. * *P*<0.05 compared with the 12 hr-time point control group. ^#^ *P*<0.05 compared with the 18 hr-time point control group. ^&&^ *P*<0.01 compared with the 24 hr-time point control group.

The mitochondria play a crucial role in cell apoptosis pathway. Loss of the mitochondrial membrane potential during apoptosis results in the release of cytochrome *c* from the mitochondria to the cytosol, which leads to induction of caspase activity and triggering of cell apoptosis. As shown in Figure 3A, treatment with physalin F caused loss of the mitochondrial membrane potential in a time-dependent manner. In addition, physalin F induced the release of cytochrome *c* into the cytosol (Fig. 3B). Furthermore, the Bcl-2 protein family regulated mitochondrial permeability and the release of cytochrome *c* to control cell apoptosis. As shown in the Figure 3C, the expression of the Bcl-2 protein family, including Bcl-2, and Bcl-xL was reduced after treatment with physalin F. However, physalin F had no significant effect on the expression of Bax (Fig. 3C). These results suggested that physalin F-induced apoptosis through a mitochondria-dependent pathway in A498 cells.

### Effects of Physalin F on Apoptosis-related Protein Expression in A498 Cells

**Figure 4:**
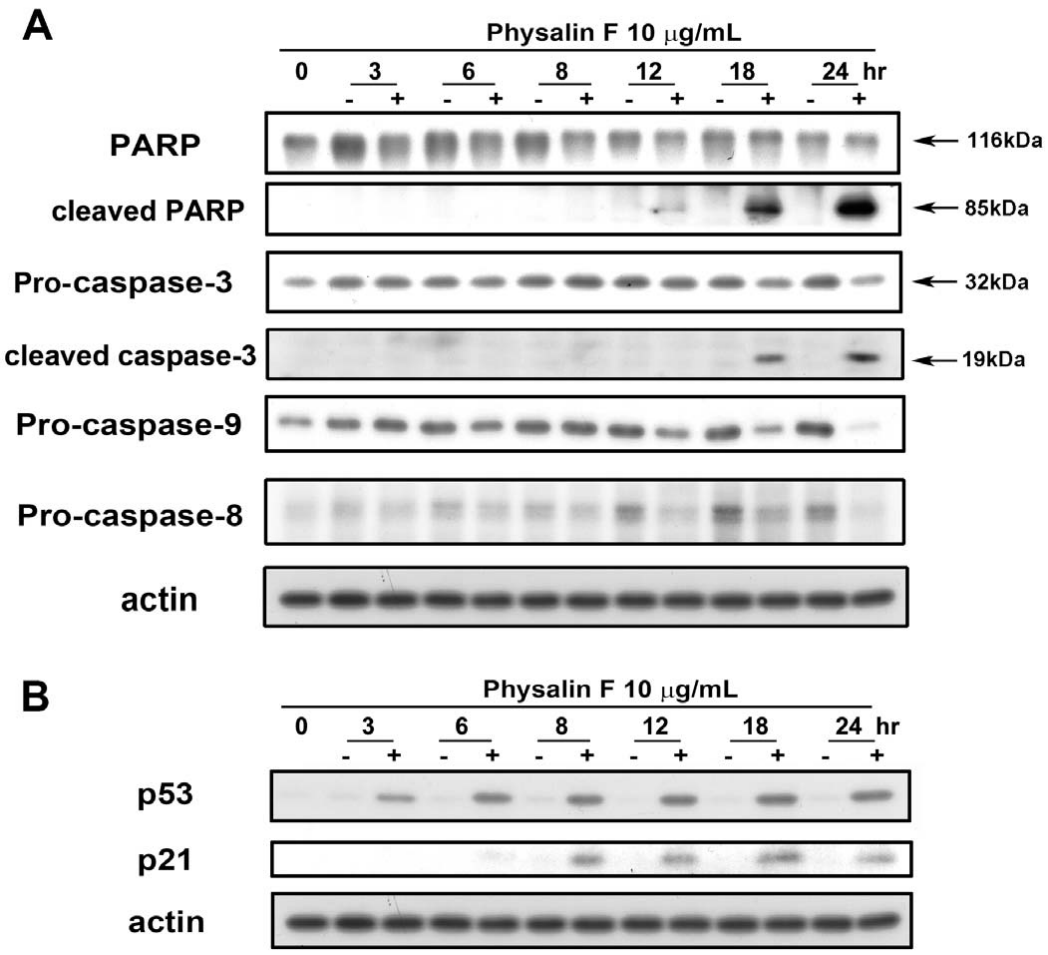
Effects of physalin F on the expression of apoptosis-related proteins in A498 cells. A498 cells were incubated in the absence or presence of physalin F for indicated time, and cells were harvested and prepared for detection of (A) pro-caspase-8, 9, caspase-3, PARP, and (B) p53, p21 by using Western blotting.

To determine the mechanism associated with Physalin F induced apoptosis, the expression of apoptosis-related proteins was investigated. Poly (ADP-ribose) polymerase (PARP) specific proteolytic cleavage, by caspases, is considered to be one of the characteristics of apoptosis; therefore, cleavage of PARP and the associated caspases were evaluated. As shown in Figure 4A, physalin F induced significant PARP and caspase-3 cleavage, accompanied by a decrease in proform levels of caspase-8 and 9.

It has been reported that p53 mediates transcriptional regulation of the cyclin-dependent kinase inhibitor p21 that plays an important role in cell apoptosis. The findings of this study showed that physalin F induced p53 and p21 in a time-dependent manner (Fig. 4B). These results indicated that physalin F-induced apoptosis via induction of p53 and p21 proteins, and subsequently cleaved caspase-8/-9/-3 and PARP.

### Physalin F-induced Cell Apoptosis through Reactive Oxygen Species Generation

**Figure 5:**
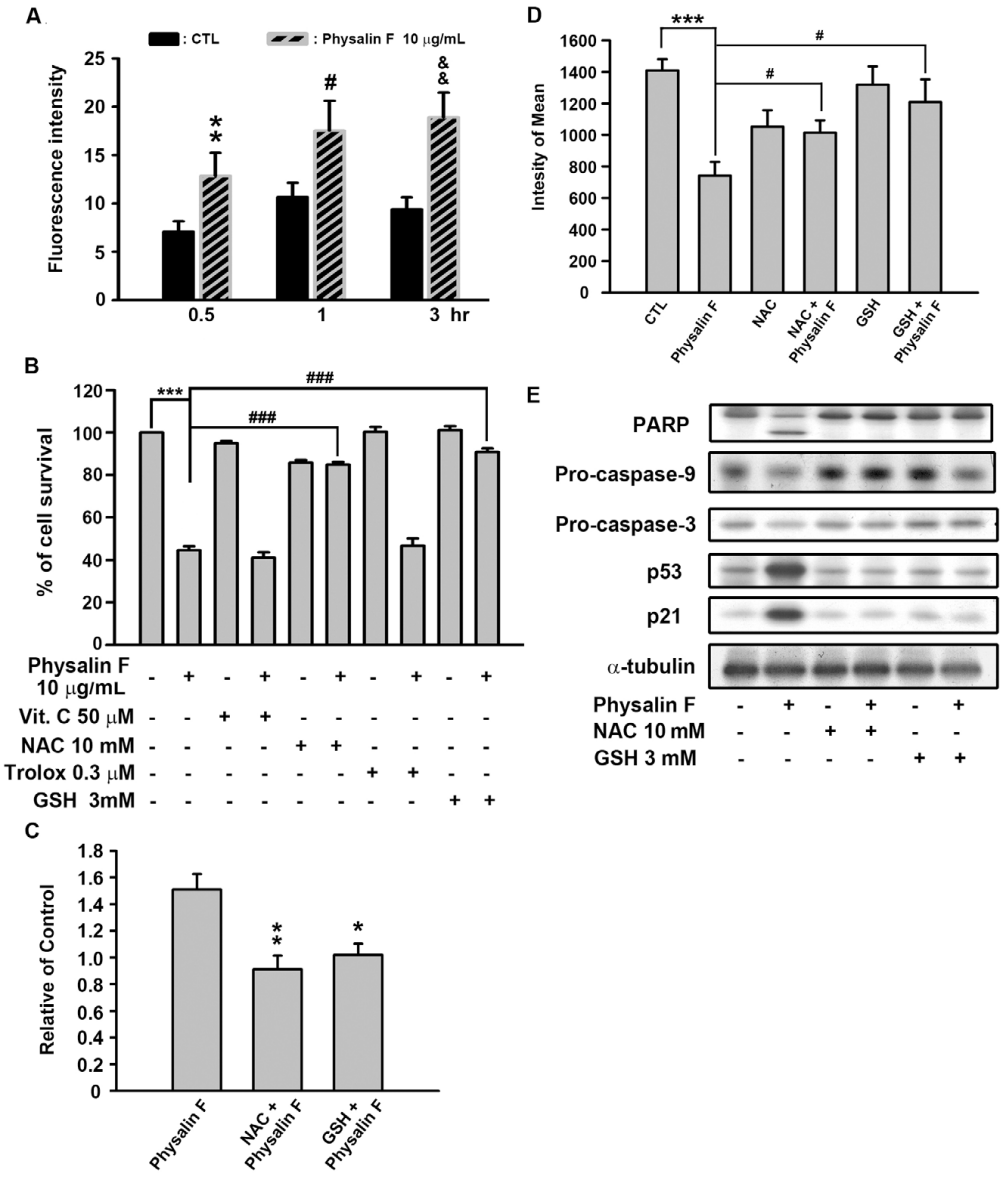
Effect of physalin F on cellular ROS accumulation in A498 cells. A498 cells were incubated in the absence or presence of physalin F (10 µg/mL) for indicated time. The fluorescent intensity of DCFH-DA was detected by flow cytometric analysis. ** *P*<0.01 compared with the 0.5 hr-time point control group. ^#^ *P*<0.05 compared with the 1 hr-time point control group. ^&&^ *P*<0.01 compared with the 3 hr-time point control group. (B) Different ROS scavengers were preincubated for 30 min and cell viability was determined by MTT assay. *** *P*<0.001 compared with the control group. ^###^ *P*<0.001 compared with the physalin F-treated group. Treatment of NAC and GSH inhibited physalin F-induced ROS generation (C, * *P*<0.05 and ** *P*<0.01 compared with the physalin F-treated group.) and the loss of Δψ_m_ (D, *** *P*<0.001 compared with the control group. ^#^ *P*<0.05 compared with the physalin F-treated group.) in A498 cells. (E) NAC (10 mM) and GSH (3 mM) reduced the apoptosis-related results in physalin F-treated cells for 24 hr.

Lee *et al.* reported that oxidative stress was involved in *Physalis angulata* extract-induced apoptosis in human oral cancer cells [23] and that the reactive oxygen species (ROS) induced mitochondrial-dependent apoptosis in many human cancer cells. In this study, physalin F-induced apoptosis mediated by ROS generation was evaluated. The results showed that ROS production was increased in a time-dependent manner after treatment with 10 µg/mL of physalin F in A498 cells (Fig. 5A). Next, four types of ROS scavengers (vitamin C, NAC, trolox, and GSH) were studied to determine which antioxidant could rescue the cells from physalin F-induced cell death. Notably, NAC and GSH significantly reversed physalin F-induced cell death according to the results of the MTT assay (Fig. 5B). These two ROS scavengers also blocked physalin F-induced ROS generation for 3 hr (Fig. 5C) and significantly reversed the physalin F-induced disruption of the mitochondrial membrane potential (Fig. 5D). In addition, the downstream signals, such as caspase-9, caspase-3, PARP, p53 and p21 were restored by NAC and GSH (Fig. 5E). These results suggested that the cell death induced by physalin F, in the A498 cells, was mediated by the accumulation of ROS and the mitochondria-dependent apoptotic pathway.

### Physalin F Inhibited NF-κB Activation

**Figure 6:**
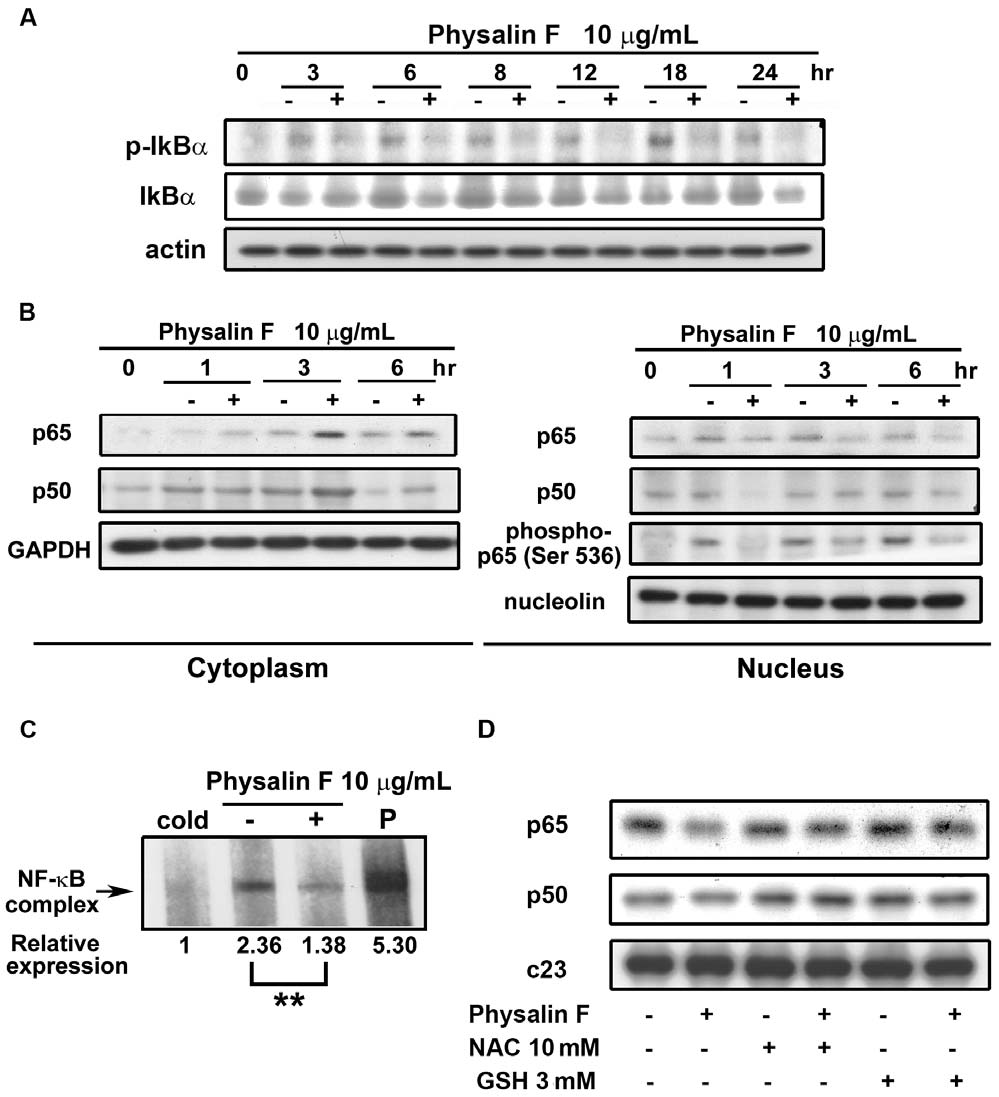
Effect of physalin F on NF-κB. A498 cells were incubated in the absence or presence of physalin F (10 µg/mL) for indicated time and detection the expression of phospho-IκBα and IκBα (A). (B) The nuclear and cytosol extract were prepared as indicated in Methods and Materials and were analyzed by Western blotting for the detection of specific protein, as indicated (p-p65, p65, p50, GAPDH, and nucleolin). (C) cells were exposed to physalin F (10 µg/mL) for 6 hr and nuclear extracts were incubated with a hot NF-κB probe (lane 2–4) or cold probe (lane 1, indicate “cold”) and demonstrate the specificity of the bands obtained on EMSA. The data are representative of three independent experiments. P indicates positive nuclear extract. ** *P*<0.01 compared with the control group. (D) Effect of ROS scavengers on physalin F-inhibited NF-κB nuclear translocation in A498 cells. Cells were pretreated with NAC (10 mM) and GSH (3 mM) for 0.5 hr and then treated with 10 µg/mL of physalin F for 6 hr. The nuclear extract were prepared as indicated in Methods and Materials. The levels of p65 and p50 were detected by Western blotting.

NF-κB, a family of transcription factors, plays a pivotal role in inflammation, immunity, angiogenesis, cell migration, cell proliferation, and apoptosis [24], [25]. In normal state, cytosolic NF-κB is bound to inhibitory IκB protein. Upon signal activation, IκBα becomes phosphorylated, ubiquitinated, and subsequentially degraded by proteasomes. The data showed that physalin F decreased the expression of IκBα and phosphorylated IκBα (Fig. 6A) and inhibited nuclear translocation of p65/p50 in A498 cells (Fig. 6B). Because phosphorylation of p65 at serine 536 by IKK is required for transcriptional activity of NF-κB, as seen in Figure 6B, we found that physalin F inhibited the phosphorylation of p65 (Ser536) in the nuclear fraction. Furthermore, to investigate whether physalin F inhibited NF-κB activity, we performed electrophoretic-mobility shift assay to observe the interaction between NF-κB and its specific recognition sequence. As shown in Figure 6C, physalin F repressed NF-κB DNA binding, and the observed signals disappeared in the presence of the cold NF-κB competitor, which indicated these signals were NF-κB specific. In addition, physalin F abrogated the nuclear translocation of p65/p50 subunits that were reversed by both ROS scavengers, NAC and GSH (Fig. 6D). These data suggested that the NF-κB/ROS pathway was involved in physalin F-induced A498 cell apoptosis.

## Discussion

Renal cell carcinoma (RCC) is the most common type of kidney cancer in adults; it is also the most deadly cancer among urological tumors. Approximately 30% of all patients with RCC will develop metastatic lesions [26]. Because of metastatic RCC (mRCC) is resistant to radiation therapy and chemotherapy, novel therapeutic treatment strategies are needed.

Physalin F is purified from *Physalis angulata* L., which has been reported to have anti-cancer activity in many cancer cell types. In this study, physalin F triggered A498 cell growth inhibition and apoptosis in a concentration- and time-dependent manner. Physalin F-induced cell apoptosis was also mediated by disruption of the mitochondria membrane potential leading to release of cytochrome *c* into the cytosol, and then activation of caspase-3/-8/-9. When cytochrome *c* is released from the mitochondria, it mediates the allosteric activation of apoptosis-protease activating factor 1 (Apaf-1). Cytochrome *c* and Apaf-1 come together to form the apoptosome. The apoptosome recruits caspase-9 and results in the proteolytic cleavage and activation of capase-9 and caspase-3 [27].

The mitochondria are the major site of production of ROS [28]. The results of this study showed that treatment with physalin F largely increased ROS production in A498 cells. ROS is critical for the metabolic and signal transduction pathways associated with cell growth and apoptosis. However, excessive production of ROS leads to oxidative stress and cell damage [29]. Therefore, the generation of ROS and pro-oxidation therapy, as a treatment for cancer, has attracted great interest. The results of this study demonstrated that physalin F-induced ROS accumulation was significantly reversed by NAC and GSH. In addition, NAC and GSH rescued the decrease of the mitochondria membrane potential and restored apoptosis-related proteins, such as capsase-9, caspase-3, PARP, p53 and p21, after physalin F treatment in A498 cells. Because NAC is a precursor of GSH, it has been reported to increase intracellular levels of glutathione, which is important to the prevention of oxidative stress in cells [30]. In addition, NAC and GSH might protect cells from damage caused by conjugation with electrophiles [30], [31]. These results show that ROS production plays an important role in physalin F-induced A498 cell apoptosis.

*Physalis angulata* L. has been commonly used as an herbal medicine to treat various inflammatory disorders, especially rheumatism, dermatitis and cancer. These observations imply that the transcriptional factor, nuclear factor-κB (NF-κB) heterodimer, plays a key role in regulating cell function after exposure to physalin F. Physalin F, extracted from *Witheringia solanacea,* was also reported to have anti-inflammatory activity by mediating NF-κB inhibition and apoptosis [32]. NF-κB is recognized to be a major transcription factor involved in cell proliferation, development, and oncogenesis. The constitutive activation of NF-κB has been reported in many human solid tumors [33]. There are emerging reports suggest that NF-κB plays a pivotal role in the development of RCC [34], [35]. Moreover, the activity of progression, invasion and metastases of RCC has been shown to correlate with the increased activation of NF-κB [36]. In our study, we examined the basal NF-κB activity in three RCC cell lines (Fig. S1). Our results showed that NF-κB was constitutive active in A498 and ACHN, but not in UO-31. In addition, physalin F is more potent in A498 cells, it suggests that inhibiting NF-κB activity by physalin F is important in A498 cells. Moreover, in our study, both of the ROS scavengers, NAC and GSH, could significantly reverse physalin F suppressed p65/p50 translocation into the nucleus, suggesting that the ROS/NF-κB pathway is involved in the cell apoptosis induced by physalin F.

Taken together, the results of this study showed that physalin F induced ROS generation and caused cell apoptosis. Because of the loss of the mitochondria membrane potential, cytochrome *c* was released into the cytosol and induced caspase activation resulting in apoptosis. In addition, NAC and GSH reversed the generation of the ROS, the disruption of mitochondria membrane potential and death of the A498 cells. Moreover, the phosphorylation of IκBα was inhibited and prevented NF-κB nuclear translocation in A498 cells with physalin F treatment. Furthermore, these phenomena were reversed by NAC and GSH. As measured by EMSA, physalin F blocked NF-κB activation in A498 cells. These findings suggest that NF-κB was involved in the physalin F-induced A498 cell apoptosis pathway. These results further the understanding of physalin F and its potential use for cancer therapy. Additional studies are needed to investigate the molecular mechanisms associated with cancer cells.

## Supporting Information

Figure S1 **NF-κB activity was evaluated by EMSA in three RCC cell lines.** The nuclear extracts of three cell lines (A498, ACHN, and UO-31 ) were incubated with a hot NF-κB probe (lane 2–4) or cold probe (lane 1, indicate “cold”) and demonstrate the specificity of the bands obtained on EMSA. P indicates positive nuclear extract.
(TIFF)
