# Supplementary figures and images for: Physalin F Induces Cell Apoptosis in Human Renal Carcinoma Cells by Targeting NF-kappaB and Generating Reactive Oxygen Species
Source: PLoS One. 2012 Jul 16;7(7):e40727. doi: 10.1371/journal.pone.0040727 (PMC3398016; doi:10.1371/journal.pone.0040727)

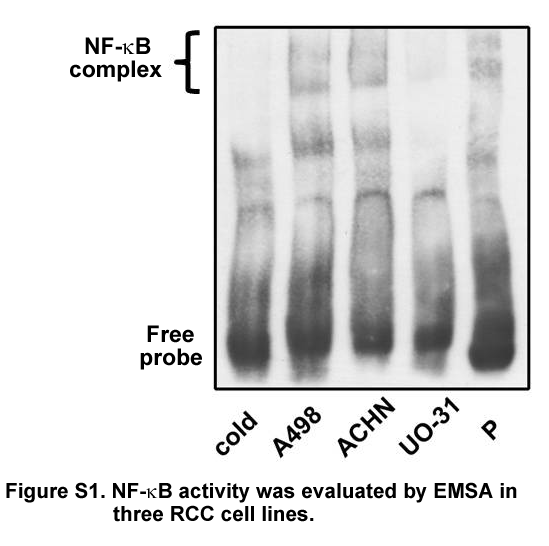

Supplement: Figure S1 — NF-κB activity was evaluated by EMSA in three RCC cell lines. The nuclear extracts of three cell lines (A498, ACHN, and UO-31 ) were incubated with a hot NF-κB probe (lane 2–4) or cold probe (lane 1, indicate “cold”) and demonstrate the specificity of the bands obtained on EMSA. P indicates positive nuclear extract. (TIFF) [file pone.0040727.s001.tiff]
